# Effects of Boxing Matches on Metabolic, Hormonal, and Inflammatory Parameters in Male Elite Boxers

**DOI:** 10.3390/medicina55060288

**Published:** 2019-06-18

**Authors:** Yakup Kılıc, Hasan N. Cetin, Esra Sumlu, Mehmet B. Pektas, Halit B. Koca, Fatma Akar

**Affiliations:** 1 Department of Coaching Education, Faculty of Sport Sciences, Fırat University, 23119 Elazığ, Turkey; yakupkilic@firat.edu.tr; 2 Department of Coaching, Faculty of Sports Science, Lokman Hekim University, 06510 Ankara, Turkey; nedim.cetin@lokmanhekim.edu.tr; 3 Department of Pharmacology, Faculty of Pharmacy, Gazi University, 06330 Ankara, Turkey; esrasmlu@gmail.com; 4 Department of Medical Pharmacology, Faculty of Medicine, Afyon Kocatepe University, 03218 Afyonkarahisar, Turkey; mbpektas@gmail.com; 5 Department of Medical Biochemistry, Faculty of Medicine, Afyon Kocatepe University, 03218 Afyonkarahisar, Turkey; bugrakoca@yahoo.com

**Keywords:** exercise, boxing match, metabolic and hormonal parameters, inflammatory markers, muscle damage indicators

## Abstract

*Background and objectives:* Boxing is a popular combat sport that requires high intensity and cooperation. However, there are limited data about the influence of boxing matches on blood parameters. The purpose of the present study was to investigate the match-induced changes in the metabolic, hormonal, and inflammatory status in male elite boxers. *Materials and methods:* High-level 20 male boxers with more than 5 years experience in boxing voluntarily participated in this study. Venous blood samples of the boxers, before and after combat, were taken for determination of the plasma parameters. *Results:* Our results indicated that a 9-min boxing match caused significant increases in plasma energy fuels (glucose and lactate), metabolic hormones (insulin, adrenocorticotropic hormone (ACTH), cortisol, and growth hormone), inflammatory markers (interleukin-1β (IL-1β), interleukin-6 (IL-6) and tumor necrosis factor alpha (TNF-α)), muscle damage indicators (alanine aminotransferase (ALT) and aspartate aminotransferase (AST)), and oxidative stress marker (SOD). A decrease in total oxidant status (TOS) was also considered. However, there were no significant alterations in the plasma levels of androgenic hormone (free and total testosterone), anabolic hormone (IGF-1), lipids (total cholesterol, triglyceride, high-density lipoprotein (HDL), and low-density lipoprotein (LDL)), kidney function markers (creatinine and urea), and minerals (iron (Fe) and magnesium (Mg)). *Conclusion:* Elevations in the level of energy fuels and metabolic hormones of the boxers could be taken as a reflection of high-energy turnover during combat performance. The increases in inflammatory and tissue damage indicators may possibly be an indication of traumatic injury. Understanding the biochemical changes that occur during boxing match could be valuable to optimize the performance improvement of the athletes.

## 1. Introduction

Boxing is a cooperative combat sport in which the competitors seek to punch their opponent without receiving a counter hit. According to the World Boxing Federation, a modern-day male boxing match consists of a determined number of rounds, each lasting 3 min divided by a 1 min interval of rest, which is characterized by short duration, high intensity, and intermittent activity. A high-level boxing match requires well-built technical, tactical, and physical skills [1,2]. In the literature, limited data are available on the effect of various combat matches on biochemical markers, hormonal status, and energy expenditure [1,3,4,5,6,7,8]. In this context, it was found that a boxing match causes an elevation in serum levels of alanine aminotransferase (ALT), aspartate aminotransferase (AST), and creatine kinase, without changing serum creatinine, in Thai boxers [3]. Fighting simulation leads to increases in cortisol and lactate, but decreases in testosterone and insulin-like growth factor-1 (IGF-1) in blood samples of Teakwando fighters [4]. A recent study demonstrated that salivary levels of testosterone and interleukin-1β (IL-1β) were decreased following a kickboxing match, whereas that of cortisol was increased [7]. The examination of the effect of performance on the hormonal and physiological status showed that the plasma levels of growth hormone, testosterone, cortisol, glucose, and lactate were raised after a simulated kickboxing match [8].

To our knowledge, there is no study of the match-induced changes on metabolic, hormonal, and inflammatory parameters of the boxers. The aim of the current study was to investigate the influence of combat matching on the biochemical status of the male elite boxers. Therefore, herein, a special focus was put on performance-induced biological changes by measuring plasma levels of glucose, lactate, insulin, adrenocorticotropic hormone (ACTH), cortisol, growth hormone, testosterone, IL-1β, interleukin-6 (IL-6), interleukin-17α (IL-17α), tumor necrosis factor alpha (TNF-α), ALT, AST, oxidative stress markers, lipid profile, urea, creatinine, and certain minerals. Understanding the biochemical changes due to a boxing match could be imperative in the maintenance of health and the improvement of athletic performance.

## 2. Materials and Methods

### 2.1. Participants

The study was undertaken in compliance with the Code of Ethics of the World Medical Association (Declaration of Helsinki) and approved by the Ethical Committee of the Dışkapı Yıldırım Beyazıt Education and Research Hospital Clinical Research (ET-18/27). The athletes gave written, informed consent after having been explained experimental procedures and possible risks of participation in the study.

### 2.2. Experimental Design

This observational research includes biochemical tests performed on blood samples from twenty active elite boxers in the age range of 20–30 years, with more than 5 years’ experience in boxing, who registered in the Turkish boxing federation. The boxers, who were eligible for the Olympic, World, or European Boxing Championship, competed in match in Turkish National Boxing Championship held in Iskenderun, 2014. Each combating match was comprised of three rounds of 3 min separated by 1 min rest. All the boxers were apparently healthy, non-smokers, and had refrained from drugs and herbal remedies categorized in WADA prohibited list. The blood collection protocol was approved with the tournament committee. Demographic and physical characteristics of boxers including age, body weight, and height were provided by the related committee.

### 2.3. Blood Collection and Analysis

To investigate metabolic, hormonal, and inflammatory parameters, the boxers were paired in proportion to weight class for fighting. The first venous blood samples (4 mL) for measurement of pre-match values of the boxers were taken 1 week before the match in order not to influence their performance. In order to determine the after-match data, the second blood samples of the boxers, who fought for 9 min without getting knocked out, were taken within 10 min at the end of the match to the BD vacutainer tubes containing lithium heparin (Becton, Dickinson and Company, Franklin Lakes, NJ, USA). After collection, the samples were centrifuged at 4 °C and 10,000× *g* for 30 min, and plasma was separated into several aliquots. The samples were stored at −85 °C until analysis.

Plasma levels of glucose (Spinreact, Girona, Spain) and lactate (Randox, Crumlin, UK) were assessed by using commercial kits. Measurement of plasma levels of insulin (DRG Instruments GmbH, Marburg, Germany), growth hormone, IGF-1, ACTH, cortisol, and total and free testosterone (Roche Diagnostic, Basel, Switzerland) were performed by using commercial ELISA kits according to the manufacturer’s instructions. Plasma levels of IL-1β, IL-6, IL-17α, and TNF-α (eBioscience, Vienna, Austria) were determined with commercial ELISA kits according to the manufacturer’s instructions. Total oxidant status (TOS) is measured using a total antioxidant status assay kit (Rel Assay diagnostics, Gaziantep, Turkey) and expressed as μmol H_2_O_2_ equivalent per liter (Equiv/L). This colorimetric assay is based on the oxidation of ferrous ion to ferric ion and the measurement of the ferric ion. Superoxide dismutase (SOD; Cayman Chemical, Ann Arbor, MI, USA); high-density lipoprotein (HDL), low-density lipoprotein (LDL), triglyceride, total cholesterol, ALT, AST, and creatinine (Spinreact, Girona, Spain); and urea, iron (Fe), and magnesium (Mg) (Roche Diagnostic, Basel, Switzerland) were determined with commercial ELISA kits according to the manufacturer’s instructions. For all assays, absorbance readings were performed with ELISA reader (ChemWell 2910, Awareness Technology, Palm City, FL, USA).

### 2.4. Statistical Analysis

Data are expressed as mean ± standard deviation (SD) or mean ± standard error mean (SEM). Graphics were drawn with Graphpad Prism 6.01 (GraphPad Software Inc., La Jolla, CA, USA). Comparisons and differences between groups were determined by using paired student’s *t*-tests. Values were considered to be significantly different when the *p* value was less than 0.05.

## 3. Results

The general physical characteristics of the boxers are presented in Table 1. The boxers’ age range was 20 to 30 years, their weight range was 49 to 110 kg, and their height range was 162 to 190 cm.

### 3.1. Metabolic Parameters

**Figure 1:**
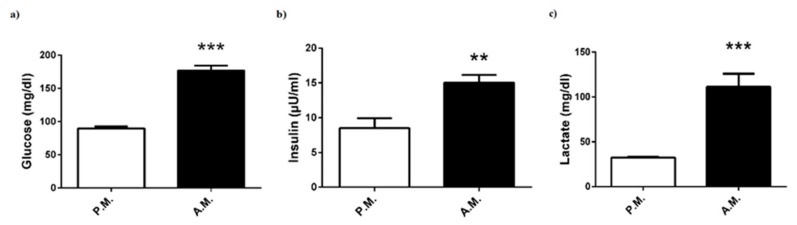
Pre-match (P.M.) and after-match (A.M.) plasma (**a**) glucose, (**b**) insulin, and (**c**) lactate levels in the boxers. The values are expressed as mean ± SEM, *n* = 20, and * *p* < 0.05 significantly different from pre-match values.

After-match levels of plasma glucose, insulin, and lactate levels of the boxers were significantly increased compared to pre-match levels (Figure 1a–c).

**Figure 2:**
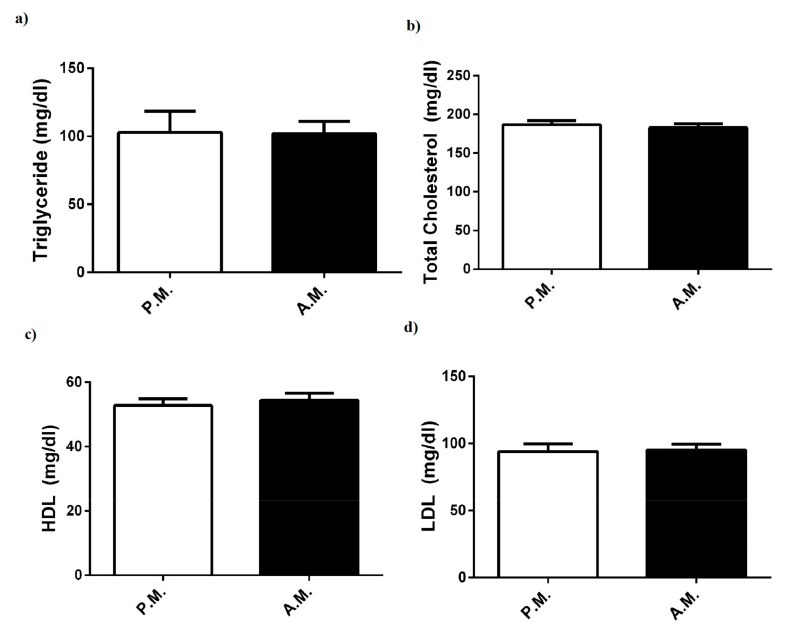
Pre-match (P.M.) and after-match (A.M.) plasma (**a**) triglyceride, (**b**) total cholesterol, (**c**) HDL, and (**d**) LDL levels in the boxers. The values are expressed as mean ± SEM, *n* = 20, and * *p* < 0.05 significantly different from pre-match values.

However, in comparison with plasma lipid profile components such as triglyceride, total cholesterol, HDL, and LDL, no significant differences were found between pre-match and after-match values of boxers (Figure 2a–d).

**Figure 3:**
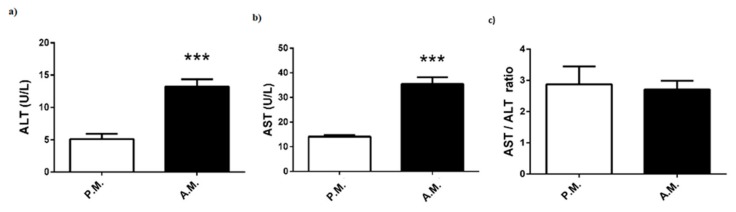
Pre-match (P.M.) and after-match (A.M.) plasma (**a**) ALT and (**b**) AST levels, and (**c**) AST/ALT ratio in the boxers. The values are expressed as mean ± SEM, *n* = 20, and * *p* < 0.05 significantly different from pre-match values.

The muscle damage markers of the boxers are reported in Figure 3. The boxing match was associated with a marked increase in ALT and AST levels (Figure 3a,b). However, AST/ALT ratio, which could be another indicator of liver or skeletal muscle injury, was the same for pre-match and after-match levels (Figure 3c).

**Figure 4:**
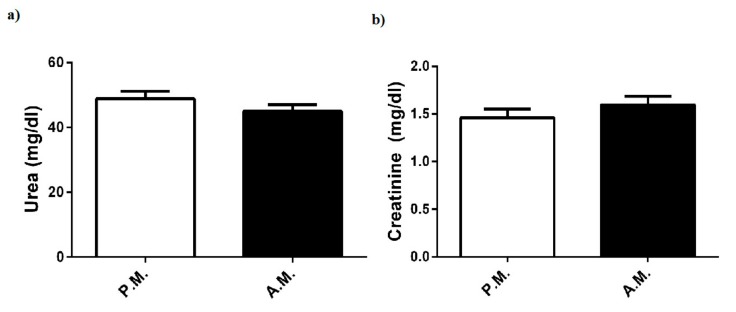
Pre-match (P.M.) and after-match (A.M.) plasma (**a**) urea and (**b**) creatinine levels in the boxers. The values are expressed as mean ± SEM, *n* = 20, and * *p* < 0.05 significantly different from pre-match values.

However, there were no changes in plasma levels of urea and creatinine, which are known as kidney function markers (Figure 4a,b).

### 3.2. Hormonal Status

**Figure 5:**
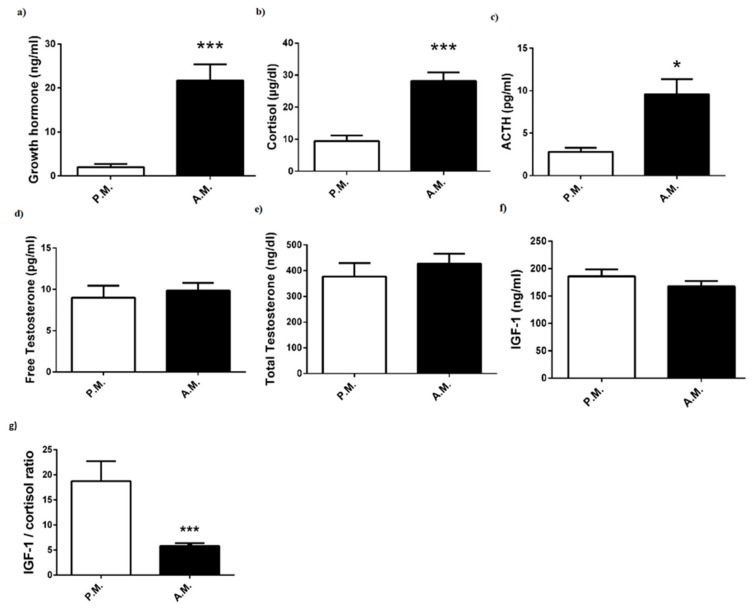
Pre-match (P.M.) and after-match (A.M.) plasma (**a**) growth hormone, (**b**) cortisol, (**c**) ACTH, (**d**) free testosterone, (**e**) total testosterone, and (**f**) IGF-1 levels; (**g**) IGF-1/cortisol ratio in the boxers. The values are expressed as mean ± SEM, *n* = 20, and * *p* < 0.05 significantly different from pre-match values.

The hormonal responses of the boxers are reported in Figure 5. After-match plasma levels of growth hormone (Figure 5a), cortisol (Figure 5b), and ACTH (Figure 5c) increased compared to pre-match levels. However, no significant alterations were found in pre-match and after-match free testosterone (Figure 5d), total testosterone (Figure 5e), and IGF-1 (Figure 5f) levels. On the other hand, after-match IGF-1/cortisol ratio was diminished compared to pre-match ratio (Figure 5g).

### 3.3. Inflammatory Parameters

**Figure 6:**
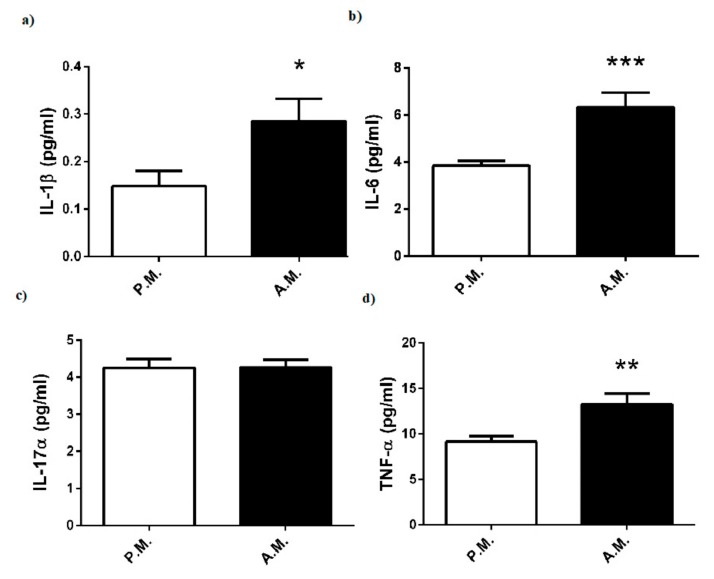
Pre-match (P.M.) and after-match (A.M.) plasma (**a**) IL-1β, (**b**) IL-6, (**c**) IL-17α, and (**d**) TNF-α levels in the boxers. The values are expressed as mean ± SEM, *n* = 20, * *p* < 0.05 significantly different from pre-match values.

The inflammatory cytokine concentrations of the boxers are reported in Figure 6. Boxing match elevated plasma levels of IL-1β (Figure 6a), IL-6 (Figure 6b), and TNF-α (Figure 6c), while it did not alter IL-17α (Figure 6d).

### 3.4. Redox Status

**Figure 7:**
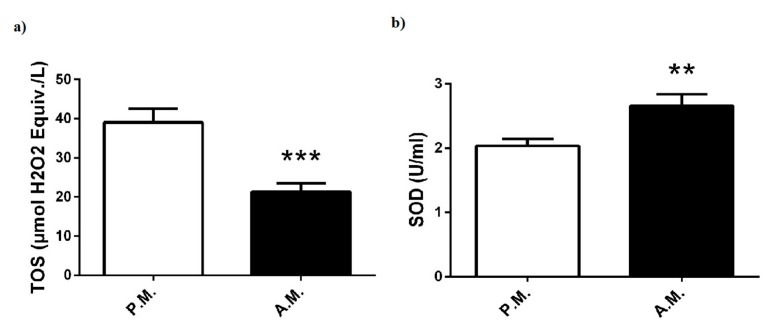
Pre-match (P.M.) and after-match (A.M.) plasma (**a**) TOS, and (**b**) SOD levels in the boxers. The values are expressed as mean ± SEM, *n* = 20, * *p* < 0.05 significantly different from pre-match values.

The oxidant and antioxidant status of boxers are given in Figure 7. Nine-minute box match led to a marked decrease in TOS levels (Figure 7a). SOD, which is another useful marker of oxidative stress, showed an increase after-match when compared to pre-match (Figure 7b).

### 3.5. Fe and Mg levels

**Figure 8:**
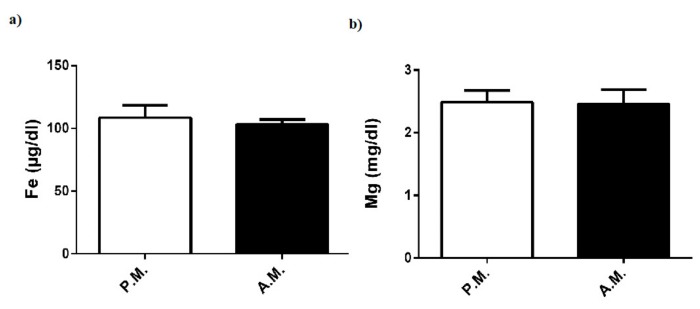
Pre-match (P.M.) and after-match (A.M.) plasma. (**a**) Fe and (**b**) Mg levels in the boxers. The values are expressed as mean ± SEM, *n* = 20, and * *p* < 0.05 significantly different from pre-match values.

Pre-match and after-match levels of plasma Fe and Mg in the boxers were the same (Figure 8a,b).

## 4. Discussion

Little data are available about the effect of boxing matches on metabolic, hormonal, and inflammatory parameters. Herein, our results indicated that a 9-min boxing match caused significant increases in plasma levels of energy fuels, metabolic hormones, and inflammatory and muscle damage indicators. The increased levels of glucose and lactate, as well as metabolic hormones, including insulin, ACTH, cortisol, and growth hormone, could be considered a reflection of metabolically high-energy production and utilization during boxing. The increases of inflammatory and tissue damage indicators reveal a traumatic injury and metabolic stress. These biological alterations during or after boxing match could be important in understanding recovery strategies and training planning of the boxers.

Several hormones affect body metabolism, in which insulin and IGF-1 exert anabolic effect by increasing glucose, amino acid, and fatty acid transport into muscles, whereas growth hormone has both catabolic and anabolic properties and cortisone has completely catabolic actions activating metabolic pathways. Physical exercise, especially high-intensity activity, leads to an increase in the level of blood glucose, which is mobilized from muscles and liver glycogen, to cover energetic demands for muscular activity, as glucose is the preferred fuel for many cells [9]. Studies on rodents and humans showed that acute exercise increased insulin-stimulated glucose transport into muscles to cover physiologically relevant requirements [10]. Sufficient insulin is necessary for entry of glucose and amino acids into cells, thereby providing fuel and essential elements for muscle activity. It has been determined that plasma insulin rises during intense exercise to regulate glucose level and restore muscle glycogen [11]. This basic knowledge will provide a reasonable explanation for our findings with a significant increase in post-match plasma glucose and insulin levels. Likewise, plasma glucose was shown to substantially increase following a kickboxing match [8]. Lactate is another energy fuel that provides maintenance of performance. Blood lactate concentrations may increase from 10- to 20-fold to 10 mmol/L in intense physical activity, because the skeletal muscle is incapable of oxidizing all of the pyruvate produced by glycolysis [9]. Previous studies have suggested that increased blood lactate concentration leads to fatigue and exhaustion. Nonetheless, recent investigations showed that high lactate levels contributed to glucose production from gluconeogenesis rather than being an anaerobic metabolic waste [12]. In simulated boxing, blood lactate concentration was shown to increase [5,8]. It was determined that post-bout blood lactate levels display an increase depending on the activity, duration, and fitness level of boxers [1,5]. Consistently, in the present study, we detected a significant increase in blood lactate concentration after the boxing bouts. Acute exercise with moderate or high intensity has been shown to elevate the secretion of growth hormone [13,14,15]. Several mechanisms have been proposed for the exercise-induced growth hormone secretion implicated in elevated lactate levels. Exercise intensity above lactate threshold initiates the secretion of growth hormone [13]. In this respect, we noticed a marked increase in both post-match plasma lactate concentration and growth hormone. Likewise, in kickboxing, blood lactate and growth hormone concentrations were shown to increase concurrently [8]. Moreover, it has been proposed that there is a possible link between secretions of growth hormone and IGF-1 during exercise. Consistently, a significant elevation in both growth hormone and IGF-1 levels following heavy resistance or acute exercise has been reported [15]. However, some studies revealed that there was a considerable discrepancy in appearance pattern of growth hormone and IGF-1, thereby pointing to the fact that IGF-1 response is independent of growth hormone secretion [13,16]. In agreement with abovementioned studies, our results indicated that circulating IGF-1 concentrations did not show any significant change following the match, despite increased plasma growth hormone concentration. Another anabolic hormone testosterone measured as free and total testosterone levels, was also not altered after the match, in conformity with the results obtained from Teakwando fighters after fighting simulation [4].

Cortisol, which is secreted in a circadian rhythm and regulated by pituitary hormone ACTH, has an important role in the maintenance of physiological homeostasis. It is generally recognized that acute or chronic exercise provokes secretion of ACTH and consequently cortisol [17]. The major function of cortisol is to provide the availability of substrates for increased metabolism during physical activity by stimulating gluconeogenesis, lipolysis, and protein catabolism. Indeed, a high blood cortisol level could be most predictive indicator of exercise-induced physical or psychological stress [18]. Herein, we measured the elevation of both plasma ACTH and cortisol concentrations after the boxing bouts thereby supporting the notion that match-induced physical or behavioral stress may lead to activation of the pituitary-adrenal axis. Of note, it is known that secretion of cortisol and ACTH shows a diurnal rhythm; however, it was not possible to perform each boxing match in the same time period. Likewise, salivary or plasma cortisol was demonstrated to increase significantly pre combat to post combat in kickboxing [6,8]. Moreover, training loads also led to an elevation in blood cortisol level of young boxers [19]. In the same study, it was also shown that the variations in IGF-1/cortisol ratio were correlated with performance and training loads [19]. This proposal is supported by our data indicating a reduction in IGF-1/cortisol ratio after the boxing bouts. The studies also suggested that there is a causal link between activation of pituitary-adrenal axis and generation of cytokines, in particular IL-6, during exercise [17,20]. IL-6 has been proposed to play a central role in the activation of cytokine cascade and regulation of energy metabolism during exercise [21,22,23]. Strenuous exercise was shown to induce an increase in the pro-inflammatory cytokines TNF-a and IL-1b, as well as a dramatic rise in anti-inflammatory cytokine IL-6, which may restrict the magnitude of the inflammatory response to exercise [22,24,25]. Consistently, in the present study, plasma levels of both pro-inflammatory cytokines (TNF-a and IL-1b) and anti-inflammatory cytokine (IL-6) were significantly increased after the boxing bouts. Our finding with IL-6 provides evidence that a counter regulatory mechanism works to limit the extent of inflammation. However, the reason for unchanged plasma level of IL-17, which is another pro-inflammatory factor, remains unknown.

Oxidative stress that occurs during exercise is another important aspect of the metabolic response [26]. Reactive oxygen species are continuously produced during cellular oxygen metabolism, and their excess generation is linked to the pathophysiology of many inflammatory diseases. A wide range of antioxidants such as SOD, catalase, and glutathione, which are produced in response to oxidative stress, minimize oxidative damage [27]. Many studies have documented that circulating level of oxidative stress markers increased after acute and high-intensity exercise [28]. However, to the best of our knowledge, there is no study that investigates the effect of box match on blood pro/antioxidant status. In the current study, TOS levels surprisingly showed a marked decrease following nine-minute boxing match. On the contrary, pre-match SOD levels increased compared to post-match SOD levels, although other antioxidant stress markers such as catalase and glutathione were not investigated in this study. The high SOD level may be a result of the restrictive effort against oxidative stress during the boxing match, thereby suggesting that its anti-oxidative capacity was sufficiently effective to overcome the short-term, match-induced oxidative stress. On the other hand, we also showed that muscle damage biomarkers such as ALT and AST were significantly increased after the boxing bouts. A tendency towards increase in blood concentration of creatinine is also possibly an indication for tissue injury. Consistent with our data, plasma ALT and AST levels were shown to rise significantly after a Thai boxing match [3]. Several investigations indicated that these parameters are increased due to muscular damage at moderate-to-high intensity physical exercise [29,30]. Thus, based on the results of the current and previous studies, we suggest that plasma ALT and AST levels may be elevated because of the damage of skeletal muscle during boxing match. This short-term, activity-induced damage might be not reflected in AST/ALT ratio.

In the current study, boxing match did not alter plasma level of renal function indicator (urea); lipid parameters (triglyceride, total cholesterol, HDL, and LDL); or certain minerals (Fe and Mg), in compliance with other combat performance findings [3,31,32]. These findings showed that the above parameters of the boxers were maintained in the normal range during high-intensity and intermittent exercise.

The study had some limitation; although we presented acute effects of boxing match on the blood parameters, the assessment of its influence at long-term periods such as 24 and 48 h after the match could be more functional to comprehend time-dependent profiles.

## 5. Conclusions

We showed that boxing match has significant effects on metabolic, inflammatory, and injury biomarkers. The boxing-induced changes may point out high-energy turnover in the company of the activation of both catabolic (ACTH and cortisol) and anabolic (insulin and growth hormone) hormonal responses, besides a traumatic injury with the provocation of inflammatory and muscle damage indicators. Furthermore, the present findings suggest that a counter regulation for biological adaptation during or after a boxing performance may occur through a catabolic and an anabolic hormonal response in the company of pro- and anti-inflammatory cytokine production. Our findings on boxing match-induced changes could be valuable for the improvement of athletic performance, training planning, and the recovery strategies of boxers.

## Figures and Tables

**Table 1:** Anthropometric characteristics of elite boxers (*n* = 20).

Parameters	Mean ± SD
Age (years)	25.88 ± 3.27
Weight (kg)	69.88 ± 15.14
Height (cm)	174.5 ± 9.71
BMI (kg/m^2^)	22.68 ± 2.70

Values are expressed as mean ± SD.
